# Association of Noncognitive Life Skills With Mortality at Middle and Older Ages in England

**DOI:** 10.1001/jamanetworkopen.2020.4808

**Published:** 2020-05-14

**Authors:** Andrew Steptoe, Sarah E. Jackson

**Affiliations:** 1 Department of Behavioural Science and Health, University College London, London, United Kingdom

## Abstract

**Importance:**

Noncognitive life skills are patterns of behavior, thoughts, and feelings that complement cognitive ability in promoting positive economic and educational outcomes. These positive attributes have been associated with favorable social and health outcomes at older ages, but their combined association with survival is not known.

**Objectives:**

To evaluate the association of the combination of 5 noncognitive life skills with mortality, and to explore the role of sociodemographic, health, cognitive, and behavioral factors in explaining associations.

**Design, Setting, and Participants:**

This cohort study used data from wave 5 of the English Longitudinal Study of Ageing, conducted in 2010. Participants included adults 52 years and older. Associations of scores on 5 noncognitive life skills, including conscientiousness, perseverance, emotional stability, optimism, and control, with all-cause mortality were analyzed for a mean (SD) of 7.2 (1.3) years. Data analyses were completed in November 2019.

**Exposures:**

Response to wave 5 of the English Longitudinal Study of Ageing.

**Main Outcomes and Measures:**

Noncognitive life skills scores, including conscientiousness, perseverance, emotional stability, optimism, and control, were measured by questionnaire. The main outcome was all-cause mortality, and the associations with noncognitive life skills scores were analyzed using Cox proportional hazards regressions models to estimate hazard ratios per 1-SD increase in score.

**Results:**

A total of 7850 participants (mean [SD] age, 66.5 [9.0] years; 4333 [55.2%] women) were included. Combined life skill score was positively associated with survival, with a hazard ratio of 0.81 (95% CI, 0.72-0.90) per 1-SD increase in positive attributes after adjustment for age, sex, race/ethnicity, childhood socioeconomic status, educational attainment, baseline chronic disease, depressive symptoms, cognitive function, mobility impairment, social isolation, smoking, physical activity, alcohol intake, and fruit and vegetable consumption (*P* < .001). Excluding deaths during 24 months after baseline as a check for reverse causation showed the same pattern (adjusted hazard ratio, 0.79; 95% CI, 0.70-0.89; *P* < .001). Associations were maintained after each life skill was omitted in turn from the aggregate score, indicating that no single positive attribute accounted for the protective association.

**Conclusions and Relevance:**

These findings suggest that noncognitive life skills are associated with survival at older ages. Whether training and education programs could enhance these attributes and influence mortality risk is not known, but fostering and maintaining life skills may be important in later life.

## Key Points

**Question:**

Are greater noncognitive life skills associated with reduced mortality in older adults?

**Findings:**

In this cohort study of 7850 adults aged 52 years and older followed up for approximately 7 years, the combination of conscientiousness, emotional stability, persistence, optimism, and sense of control was associated with reduced mortality independently of sociodemographic, health, and behavioral factors, but no single life skill explained the association.

**Meaning:**

These findings suggest that higher levels of noncognitive life skills are associated with longer survival, suggesting that maintenance of these skills in later life is relevant to health.

## Teaser

This cohort study examines the associations of noncognitive life skills, including conscientiousness, emotional stability, persistence, optimism and sense of control, with mortality in adults 52 years and older.

## Introduction

Noncognitive life skills are patterns of behavior, thoughts, and feelings, such as perseverance, self-control, conscientiousness, social skills, and emotional stability. They have come into prominence in economic and policy research as positive psychological attributes that contrast with cognitive skills or ability. There is substantial evidence that they are important in promoting future economic, educational, and prosocial outcomes in early life.^1,2,3,4^ Some of these characteristics can be regarded as aspects of personality, although the term *skill* has been used to highlight the notion that they are malleable rather than fixed traits. The accumulation of noncognitive life skills in later life seems to be relevant as well. An analysis of more than 8000 participants in the English Longitudinal Study of Ageing (ELSA) measured 5 positive attributes, including conscientiousness, emotional stability, persistence or determination, optimism, and sense of control, and demonstrated that aggregate life skills were associated with favorable economic, social, health, and biological outcomes.^5^ The combination of life skills was also associated with less depression and loneliness, a lower incidence of chronic disease and disability, and better physical capability 4 years later, even after taking baseline function into account. These findings from older people in England were replicated in the Health and Retirement Study in the US, where longitudinal associations were also observed between number of life skills and less anxiety and chronic stress and more prosocial behavior.^6^ No single life skill was responsible for these findings, and associations were independent of cognitive ability, education, and childhood socioeconomic advantage.

The association of combined noncognitive life skills with survival has not been investigated extensively, to our knowledge. Individual components, such as conscientiousness, emotional stability, optimism, and sense of control, have been associated with reduced mortality,^7,8,9,10^ but it is not known whether the combination of these positive attributes is associated with longer survival. Accordingly, we assessed all-cause mortality during an approximately 7-year follow-up period in the ELSA sample in which other outcomes have previously been investigated.^5^ In addition to evaluating the combination of noncognitive life skills, we tested whether any particular skill was especially associated with survival. We also assessed the extent to which socioeconomic factors, chronic physical illness, depression, mobility impairment, cognitive ability, social isolation, and health behaviors explained the associations of noncognitive life skills with survival.

## Methods

### Study Sample

The ELSA is a longitudinal panel study of men and women 50 years and older living in England that started in 2002.^11^ The methods of data collection and questionnaires have been published previously.^12^ The data used for our analyses were collected in wave 5 of ELSA in 2010, since this was the first occasion on which data for several of the noncognitive life skills were obtained. Data from ELSA can be accessed from the UK Data Service.^13^ The study was conducted in accordance with the World Medical Association Declaration of Helsinki.^14^ The ELSA study was approved by the UK’s National Research Ethics Service, and all participants provided informed consent in person. This report follows the Strengthening the Reporting of Observational Studies in Epidemiology (STROBE) reporting guideline. Data analyses were completed in November 2019.

### Measures

#### Noncognitive Life Skills

Scales from the Midlife Development Inventory Personality Scales^15^ were used to measure conscientiousness and emotional stability. These scales have been used in several previous analyses of the Midlife in the United States study and the Health and Retirement Study. Participants were asked the extent to which each of 26 adjectives described themselves on a scale ranging from 1, indicating not at all, to 4, a lot. Four items (eg, organized, responsible) contributed to the conscientiousness scale, and 6 items (eg, moody, worrying) to the emotional stability scale. Persistence was assessed with a single rating of the extent to which participants had felt determined during the past 30 days (responses ranged from not at all to very much). Optimism was measured with ratings of 2 statements “I feel that life is full of opportunities” and “I feel that the future looks good for me,” while sense of control was indexed by the single item “At home, I feel I have control over what happens in most situations.” An aggregated index of noncognitive life skills was created by standardizing ratings for each skill (mean [SD], 0 [1.0]), and deriving the mean of standardized ratings. We also created aggregated life skill scores omitting each of the 5 capabilities in turn to assess whether any single skill accounted for associations with mortality.

#### Mortality

The main outcome, all-cause mortality, was assessed up to February 2018. Mortality data were obtained through linkage with the National Health Service central data registry for all participants who consented to mortality follow-up, indicating death by year and month.

#### Covariates

We included as covariates demographic variables together with factors that might be associated with noncognitive life skills and mortality risk. Age at baseline (ie, in 2010) was classified into 4 categories: 52 to 59 years, 60 to 69 years, 70 to 79 years, and 80 years or older. Race/ethnicity was categorized as white or other and was self-reported. Childhood socioeconomic status (SES) was indexed by the occupation of the participant’s father or main caregiver when they were aged 14 years, divided into routine, intermediate, and managerial or professional. Education was measured as the participant’s highest educational qualification and divided into 4 categories: basic education (ie, no qualifications), some junior high school qualifications (ie, O levels, approximately equivalent to US 7th or 8th grades), high school graduation (ie, A levels, approximately equivalent to US 12th grade), and college or university education. Chronic physical illness at baseline was indicated by summing the number of diagnosed serious diseases, including coronary heart disease, cancer, stroke, diabetes, arthritis, and chronic lung disease. Depression was assessed with symptoms measured using the 8-item Center for Epidemiologic Studies Depression Scale, with a score of 4 or higher being used to indicate significant symptoms, as used previously in ELSA analyses.^16^ Cognitive capacity was measured by aggregating performance on 5 objective tests administered in face-to-face interviews. These were immediate recall, delayed recall, verbal fluency, and speed and accuracy on a letter cancellation task. We *z*-transformed scores on the 5 tests and found the mean to generate an index of cognitive function. Mobility impairment was assessed by summing the presence or absence of 10 mobility issues (eg, walking 100 yards, reaching or extending arms above shoulder level). Social isolation was assessed using a measure of the extent of contact with children, other family members, and friends and participation in organizations and clubs.^17^ Four activities were included in the measure of health behavior: smoking, physical activity (a 5-level categorization detailed elsewhere^18^), number of units of alcohol consumed per week, and number of portions of fruit and vegetables eaten daily.

### Statistical Analysis

Associations between the index of noncognitive life skills and mortality were analyzed using Cox proportional hazards regression models, estimating hazard ratios (HRs) and 95% CIs per 1-SD increase in score. Checking Schoenfeld residuals indicated that proportional hazards assumptions were met in the Cox regression models. Survival time was measured in months from baseline (ie, the date of the wave 5 interview) to death or March 2018, whichever came first. We computed 2 models: a basic model adjusting for age and sex, and a fully adjusted model adjusting for age, sex, race/ethnicity, childhood SES, educational attainment, chronic disease, depressive symptoms, cognitive function, mobility impairment, social isolation, smoking, physical activity, fruit and vegetable consumption, and alcohol intake. Observational studies are liable to confounding by unmeasured variables. As a check for this possibility, *E*-values were calculated for HRs to estimate the strength of association an unmeasured confounder would need to have with both life skills and mortality to explain the positive attribute–mortality associations.^19,20^ The possibility of reverse causality (ie, terminal illness leading to reduced noncognitive life skills) was addressed by excluding deaths occurring within 24 months of baseline. The importance of individual life skills in association with mortality was tested by removing each in turn from the aggregated index and calculating the proportion of the protective association that remained after that component was removed.

We also tested the extent to which different factors explained the associations between noncognitive life skills and mortality by testing 7 sets of potential mediators and confounders: sociodemographic factors (ie, race/ethnicity, childhood SES, and educational attainment), baseline physical health (ie, number of chronic diseases), depressive symptoms, cognitive function, mobility impairment, social isolation, and health behavior (ie, smoking, physical activity, fruit and vegetable consumption, and alcohol intake). In each case, we computed HRs for noncognitive life skills when including the potential mediator in the model, and computed the percentage of the protective association explained (PPAE) using the formula PPAE = (HR [adjusted for noncognitive life skills, age, sex, race/ethnicity, childhood SES, education, and mediator] – HR [adjusted for noncognitive life skills, age, sex, race/ethnicity, childhood SES, and education]) / (1 − HR [adjusted for noncognitive life skills, age, sex, race/ethnicity, childhood SES, and education]) × 100.^21^ This method has been widely used in epidemiological studies.^22^

## Results

Of 8119 adults aged 52 years and older assessed in 2010 and analyzed in our previous study,^5^ 8117 (99.9%) were tracked until March 2018, a mean follow-up of 7.2 [1.3] years. Data were missing on some covariates for 267 individuals, so the analytic sample included 7850 individuals (mean [SD] age, 66.5 [9.0] years; 4333 [55.2%] women). Table 1 summarizes the characteristics of the study sample. Participants were predominantly white (7673 individuals [97.7%]). There were 1030 deaths (13.1%) during the follow-up period. The univariable associations between sample characteristics and mortality are detailed in eTable 1 in the Supplement. Death was more common among men than women (549 men [15.6%] vs 481 women [11.1%]; *P* < .001), and was associated with greater age (age 52-59 years: 63 deaths among 2003 individuals [3.1%]; age 60-69 years: 205 deaths among 3125 individuals [6.6%]; age 70-79 years: 405 deaths among 1990 individuals [20.4%]; age ≥80 years: 357 deaths among 732 individuals [48.8%]; *P* < .001), lower childhood SES (routine: 371 deaths among 2484 individuals [14.9%]; intermediate: 435 deaths among 3308 individuals [13.6%]; managerial or professional: 224 deaths among 2158 individuals [10.4%]; *P* < .001), less education (no qualifications: 384 deaths among 1827 individuals [21.0%]; up to O level: 234 deaths among 875 individuals [12.5%]; A level or equivalent: 146 deaths among 1249 individuals [11.7%]; college or university: 266 deaths among 2899 individuals [9.2%]; *P* < .001), more chronic diseases (none: 246 deaths among 3445 individuals [7.1%]; 1 chronic disease: 393 deaths among 2948 individuals [13.3%]; 2 chronic diseases: 253 deaths among 1069 individuals [23.7%]; 3 chronic diseases: 111 deaths among 320 individuals [34.7%]; 4 chronic diseases: 19 deaths among 55 individuals [34.5%]; ≥5 chronic diseases: 8 deaths among 13 individuals [61.5%]; *P* < .001), depressive symptoms (no symptoms: 802 deaths among 6822 individuals [11.8%]; significant symptoms: 228 deaths among 1028 individuals [22.2%]; *P* < .001), social isolation (not isolated: 536 deaths among 4450 individuals [12.0%]; isolated: 494 deaths among 3400 individuals [14.5%]; *P* < .001), and smoking (no smoking: 866 deaths among 6907 individuals [12.5%]; current smoking: 164 deaths among 943 individuals [17.4%]; *P* < .001). Mortality risk was reduced in participants with higher cognitive scores (*r* = −0.224; *P* < .001), more physical activity (*r* = −0.234; *P* < .001), and greater fruit and vegetable consumption (*r* = −0.059; *P* < .001) and increased for individuals with mobility impairment (*r* = 0.215; *P* < .001).

**Table 1.** Characteristics of the Analytic Sample (n = 7850)

Characteristic	No. (%)
Sex	
Men	3517 (44.8)
Women	4333 (55.2)
Age, y	
52-59	2003 (25.5)
60-69	3125 (39.8)
70-79	1990 (25.4)
≥80	732 (9.3)
Race/ethnicity^a^	
White	7673 (97.7)
Other	177 (2.3)
Noncognitive life skill score, mean (SD)^b^	
Conscientiousness	2.30 (0.49)
Emotional stability	1.60 (0.47)
Persistence	3.67 (0.98)
Optimism	3.10 (0.75)
Control	5.18 (0.93)
Childhood SES	
Routine	2484 (31.6)
Intermediate	3208 (40.9)
Managerial professional	2158 (27.5)
Education	
No qualifications	1827 (23.3)
Up to O level	1875 (23.9)
A level or equivalent	1249 (15.9)
College or university	2899 (36.9)
Chronic diseases, mean (SD), No.	0.81 (0.89)
Depressive symptoms^c^	
No symptoms	6822 (86.9)
Significant symptoms	1028 (13.1)
Cognition, standardized score, mean (SD)	0.028 (0.64)
Mobility impairments, mean (SD), No.	1.79 (2.43)
Social isolation^a^	
Not isolated	4450 (56.7)
Some isolation	3400 (43.3)
Smoking	
Nonsmoker	6907 (88.0)
Current smoker	943 (12.0)
Physical activity score, mean (SD)^a^	2.18 (1.31)
Alcohol consumption, mean (SD), units/wk^d^	2.86 (5.88)
Fruit and vegetables, mean (SD), portions/d^e^	5.05 (2.47)

^a^ Includes data for 8116 individuals.

^b^ Scales for individual domains are conscientiousness, 0-3; emotional stability, 0-3; persistence, 1-5; optimism, 1-4; and control, 1-6.

^c^ Includes data for 8056 individuals.

^d^ Includes data for 8073 individuals.

^e^ Includes data for 7952 individuals.

Results of the Cox proportional hazards regression analyses of the association of aggregate noncognitive life skills with all-cause mortality are shown in Table 2. There was a significant protective association in the basic age-and sex-adjusted model, with an HR of 0.58 (95% CI, 0.53-0.63; *P* < .001). The association was reduced in the fully-adjusted model but remained statistically significant, with an HR of 0.81 (95% CI, 0.72-0.90; *P* < .001), indicating a 19% reduction of hazard of dying for every 1-SD increase in noncognitive life skills, after controlling for sociodemographic, health, social, and lifestyle factors. In the full regression model, mortality was independently associated with sex (adjusted HR for women compared with men, 0.59; 95% CI, 0.51-0.67; *P* < .001), greater age (adjusted HR compared with age 52-59 years: age 60-69 years, 1.97; 95 % CI, 1.49-2.62; age 70-79 years, 5.43; 95 % CI, 4.13-7.15; age ≥80 years, 12.80; 95 % CI, 9.61-17.04; *P* < .001), baseline chronic disease (adjusted HR, 1.16; 95% CI, 1.08-1.23; *P* < .001), lower cognition (adjusted HR, 0.76; 95% CI, 0.69-0.84; *P* < .001), smoking (adjusted HR, 1.64; 95% CI, 1.37-1.96; *P* < .001) and less physical activity (adjusted HR, 0.81; 95% CI, 0.77-0.86; *P* < .001) (eTable 2 in the Supplement). The *E*-value for the fully adjusted model was 1.77 (lower 95% CI, 1.46) (Table 2).

**Table 2.** Association of Combined Noncognitive Life Skills With Mortality

Model	Hazard ratio (95% CI)	*P* value	E-value (lower 95% CI)
Basic^a^	0.58 (0.53-0.63)	<.001	2.84 (2.55)
Fully adjusted^b^	0.81 (0.72-0.90)	<.001	1.77 (1.46)
Excluding deaths in first 24 mo after baseline			
Basic^a^	0.59 (0.54-0.66)	<.001	2.78 (2.40)
Fully adjusted^b^	0.79 (0.70-0.89)	<.001	1.85 (1.50)

^a^ Adjusted for age and sex.

^b^ Adjusted for age, sex, ethnicity, childhood socioeconomic status, educational attainment, chronic disease, depressive symptoms, cognitive function, mobility impairment, social isolation, smoking, physical activity, fruit and vegetable consumption, and alcohol intake.

We tested for reverse causality by excluding all deaths occurring within 24 months of the measurement of noncognitive life skills, reasoning that participants might have had serious illnesses leading to death soon after baseline that reduced life skill scores, resulting in reverse causation among those experiencing early death. However, the association between noncognitive life skills and mortality was maintained (Table 2), with a 21% reduction in hazard for every 1-SD increase in life skills in the fully adjusted model (adjusted HR, 0.79; 95% CI, 0.70-0.89; *P* < .001).

Since several of noncognitive life skills have previously been shown to be associated with survival, we tested whether a particular factor was responsible for the association of the aggregate life skill index with survival. Each component was therefore omitted from the combined index in turn, and the associations of the remaining factors with survival were calculated. The results indicate that the associations of noncognitive life skills with survival were slightly reduced after each component was removed in turn, but nonetheless remained significant in each case (conscientiousness: adjusted HR, 0.86; 95% CI, 0.77-0.95; *P* < .001; emotional stability: adjusted HR, 0.83; 95% CI, 0.76-0.90; *P* < .001; persistence: adjusted HR, 0.81; 95% CI, 0.73-0.90; *P* < .001; optimism: adjusted HR, 0.83; 95% CI, 0.75-0.92; *P* < .001; control: adjusted HR, 0.80; 95% CI, 0.72-0.90; *P* < .001) (Table 3).

**Table 3.** Association of Combined Noncognitive Life Skills With Mortality After Removing Each Component

Component excluded	Adjusted hazard ratio (95% CI) ^a^	*P* value	E-value (lower 95% CI)
None	0.81 (0.72-0.90)	<.001	1.77 (1.46)
Conscientiousness	0.86 (0.77-0.95)	<.001	1.60 (1.29)
Emotional stability	0.83 (0.76-0.90)	<.001	1.70 (1.46)
Persistence	0.81 (0.73-0.90)	<.001	1.77 (1.46)
Optimism	0.83 (0.75-0.92)	<.001	1.70 (1.39)
Control	0.80 (0.72-0.90)	<.001	1.81 (1.46)

^a^ Fully adjusted for age, sex, ethnicity, childhood socioeconomic status, educational attainment, chronic disease, depressive symptoms, cognitive function, mobility impairment, social isolation, smoking, physical activity, fruit and vegetable consumption, and alcohol intake.

Table 4 summarizes the role of 7 sets of potential mediators and confounders in explaining the association of combined noncognitive life skills with survival. The associations of life skills with survival remained significant after each potential mediator or confounder was added to the model. The importance of each set of factors ranged from social isolation, which explained only 2% of the association of noncognitive life skills with survival, to sociodemographic factors (PPAE, 26%), health behaviors (PPAE, 26%), and mobility impairment (PPAE, 24%). Nonetheless, all factors together explained only approximately 55% of the association of noncognitive life skills with survival, implying that almost half of the association was not explained by these factors.

**Table 4.** Association of Combined Noncognitive Life Skills and Potential Mediators and Confounders With Mortality

Variable	Adjusted hazard ratio (95% CI)^a^	*P* value	PPAE, %	*E*-value (lower 95% CI)
Basic model	0.58 (0.53-0.63)	<.001	NA	2.84 (2.55)
Potential mediators or confounders				
Sociodemographic factors	0.69 (0.58-0.82)	<.001	26	2.26 (1.74)
Chronic disease	0.62 (0.57-0.68)	<.001	14	2.61 (2.30)
Depression	0.64 (0.58-0.71)	<.001	10	2.50 (2.17)
Cognition	0.62 (0.57-0.68)	<.001	10	2.61 (2.30)
Mobility impairment	0.68 (0.62-0.75)	<.001	24	2.30 (2.00)
Social isolation	0.59 (0.53-0.64)	<.001	2	2.28 (2.50)
Health behaviors	0.69 (0.63-0.76)	<.001	26	2.26 (1.96)
All	0.81 (0.72-0.90)	<.001	55	1.77 (1.46)

Abbreviations: NA, not applicable; PPAE, percentage of protective association explained.

^a^ Adjusted for age and sex.

## Discussion

The findings of this cohort study suggest that the combination of noncognitive life skills at older ages was associated with reduced all-cause mortality in a representative population sample. No single life skill explained this association, since the association with survival remained significant after each component was omitted in turn. The results indicated that approximately half the association of noncognitive life skills and survival was explained by the various mediators studied, including sociodemographic factors (eg, early life SES and educational attainment), baseline health, depressive symptoms, mobility, cognition, social isolation, and health behaviors. Nevertheless, even when all these factors were taken into account, individuals with high scores in noncognitive life skills had a 19% reduction in risk of dying during the 7-year follow-up period compared with those with lower noncognitive life skill scores. The *E*-values suggest that the associations are probably robust to unmeasured confounders. There is no absolute level for *E*-values above which confounding is definitely ruled out.^20^ However, the *E*-value of 1.77 in the primary analysis indicates that an unmeasured confounder would have to have a relative risk association with both noncognitive life skills and mortality of at least 1.77 to explain the association. Considering that smoking had an HR of 1.64 (95% CI, 1.37-1.96) for mortality, the unmeasured confounder would need to have a greater association even than smoking, and this seems unlikely.

Nomenclature in this field of research is problematic. Some, but not all, of the noncognitive life skills are personality factors. The term *noncognitive life skill* was introduced by researchers from economic and public policy disciplines to distinguish these attributes from cognitive skills and to emphasize that they are modifiable. Other authorities have labeled these factors as aspects of personality or character.^23^ We used the term *noncognitive life skill* to encompass the range of phenomena under investigation and to distinguish these factors from features of positive well-being, such as happiness or sense of purpose. There is increasing evidence from epidemiology and personality psychology that individual positive attributes are associated with survival, following from the pioneering work of Thomas and McCabe,^24^ Vaillant,^25^ and others. Thus, large population studies have demonstrated that individuals high in conscientiousness and emotional stability are at reduced risk of mortality.^7,8,26,27^ Greater optimism is associated with reduced all-cause and cardiovascular mortality,^10,28^ while greater control also has protective associations.^9,29^ Few studies have examined combinations of these factors, although some investigators have assessed whether associations are independent of each other.^7,8^ Our study adds to the literature by showing the relevance of noncognitive life skills in combination. It is interesting that no single component drove the findings, supporting the argument that the accumulation of these life skills is relevant to healthy aging. Noncognitive life skills are important not only for survival but also for social, economic, and mental health outcomes.^5,6^

We evaluated the processes potentially responsible for explaining the associations of noncognitive life skills with survival by studying 7 sets of factors, some of which could potentially act as confounders (eg, education, chronic disease) whereas others could be mediators (eg, social isolation, health behaviors). In addition to sociodemographic factors, such as education and childhood SES, 2 factors were particularly important. First, mobility impairment accounted for 24% of the protective association of noncognitive life skills with mortality. We do not know whether lower life skills earlier in life determined in part the development of impaired mobility, or whether mobility deficits led to reductions in noncognitive skills, such as optimism and persistence. Second, health behaviors, including smoking, physical inactivity, excessive alcohol consumption, and poor diet, explained 26% of the association. Previous studies have shown that the association of emotional stability (but not conscientiousness) with mortality is partly mediated by smoking,^7^ and our results suggest that a broader set of health behaviors is relevant.

Nevertheless, a substantial proportion of the association of noncognitive life skills with mortality was not explained by mobility, health behavior, demographic factors, cognitive function, the presence of serious illness at baseline, depressive symptoms, or social isolation. Additional pathways must therefore be in part responsible. Unmeasured behaviors may be relevant, including adherence to medical advice or medication, taking opportunities for screening for serious diseases, or responding promptly to medical symptoms.^30^ Direct influences on biological processes contributing to health risk, such as cardiovascular and metabolic regulation, inflammatory responses, and neuroendocrine pathways, may also contribute. For example, high levels of conscientiousness, emotional stability, and greater optimism have been associated with reduced cortisol output and inflammation.^31,32,33,34^

### Limitations

This study had some limitations. First, we were limited by the noncognitive life skills assessed in ELSA. Therefore, we were not able to include measures of social competence and social skill, coping, or other elements that are prominent in the noncognitive life skill literature.^2,4^ Some measures of life skills were suboptimal. For example, optimism was assessed with 2 items, although similar results were obtained in the Health and Retirement Study when optimism was quantified with a standard questionnaire.^6^ We do not know whether the life skills assessed in this study reflect capabilities maintained since early in life or were developed more recently. Life course studies are needed to investigate this issue, since the youngest participants in ELSA were in their 50s. Research involving more diverse cohorts in terms of age, race/ethnicity, and geography are desirable to help gauge the generalizability of the findings.

## Conclusions

The results of this cohort study add weight to the importance of noncognitive life skills at older ages by documenting associations with survival. Training and education programs may be relevant even in middle and older age, but evaluating the effects of intervention on longevity is a major challenge.

## Notes

Supplement. **eTable 1.** Univariable Associations of Covariates With Mortality **eTable 2.** Full Regression Model for Complete Sample
