# Supplementary material for: Association of Noncognitive Life Skills With Mortality at Middle and Older Ages in England
Source: JAMA Netw Open. 2020 May 14;3(5):e204808. doi: 10.1001/jamanetworkopen.2020.4808 (PMC7225907; doi:10.1001/jamanetworkopen.2020.4808)
Supplement: Supplement. — eTable 1. Univariable Associations of Covariates With Mortality eTable 2. Full Regression Model for Complete Sample [file jamanetwopen-3-e204808-s001.pdf]

Supplementary Online Content

Steptoe A, Jackson SE. Association of noncognitive life skills with mortality at middle and older ages in England. *JAMA Netw Open*. 2020;3(5):e204808.
doi:10.1001/jamanetworkopen.2020.4808

eTable 1. Univariable Associations of Covariates With Mortality

eTable 2. Full Regression Model for Complete Sample

This supplementary material has been provided by the authors to give readers additional information about their work.

© 2020 Steptoe A et al. *JAMA Network Open.*

eTable 1. Univariable Associations of Covariates With Mortality

**Variable**	**Category**	**Fatalities/ Sample at risk (N)**	**Fatalities (%)**	**X^2 / *r***	***P***
Sex	Men<br>Women	549 / 3517<br>481 / 4333	15.6%<br>11.1%	34.42	<0.001
Age (y)	52-59<br>60-69<br>70-79<br>80+	63 / 2003<br>205 / 3125<br>405 / 1990<br>357 / 732	3.1%<br>6.6%<br>20.4%<br>48.8%	1004.08	<0.001
Ethnicity	White<br>Non-white	1013 / 7673<br>18 / 177	13.2%<br>9.6%	2.13	0.17
Childhood SES	Routine<br>Intermediate<br>Managerial / professional	371 / 2484<br>435 / 3308<br>224 / 2158	14.9%<br>13.6%<br>10.4%	20.59	<0.001
Education	No qualifications<br>Up to O level<br>A level or equivalent<br>College or University	384 / 1827<br>234 / 1875<br>146 / 1249<br>266 / 2899	21.0%<br>12.5%<br>11.7%<br>9.2%	133.43	<0.001
Chronic disease (n)	0<br>1<br>2<br>3<br>4<br>5+	246 / 3445<br>393 / 2948<br>253 / 1069<br>111 / 320<br>19 / 55<br>8 / 13	7.1%<br>13.3%<br>23.7%<br>34.7%<br>34.5%<br>61.5%	377.64	<0.001
Depressive symptoms	No symptoms<br>Significant symptoms	802 / 6822<br>228 / 1028	11.8%<br>22.2%	74.70	<0.001
Cognition	Standardized score			*r* = -0.224	<0.001
Mobility impairments	N impairments			*r* = 0.215	<0.001
Social isolation	Not isolated<br>Some isolation	536 / 4450<br>494 / 3400	12.0%<br>14.5%	10.38	<0.001
Smoking	Non-smoker<br>Current smoker	866 / 6907<br>164 / 943	12.5%<br>17.4%	15.98	<0.001
Physical activity	1 -5 rating			*r* = -0.234	<0.001

© 2020 Steptoe A et al. *JAMA Network Open.*

Alcohol consumption	N units / week			*r* = -0.016	0.15
Fruit and vegetables	N portions / day			*r* = -0.059	<0.001

X^2 analysis for categorical variables, and point biserial correlations (r) for continuous variables

© 2020 Steptoe A et al. *JAMA Network Open.*

eTable 2. Full Regression Model for Complete Sample

**Variable**	**Adjusted HR (95% CI)**	**P**
Non-cognitive life skills	0.81 (0.72 – 0.90)	<0.001
Sex (male reference)	0.59 (0.51 – 0.67)	<0.001
Age		
52-59	1	
60-69	1.97 (1.49 – 2.62)	<0.001
70-79	5.43 (4.13 – 7.15)	<0.001
80+	12.80 (9.61 – 17.04)	<0.001
Ethnicity (white reference)	0.85 (0.52 – 1.38)	0.51
Childhood SES		
Routine	1	
Intermediate	0.99 (0.86 – 1.14)	0.87
Managerial / professional	1.05 (0.88 – 1.25)	0.81
Education		
No qualifications	1	
Up to O level	1.0 (0.84 – 1.18)	0.99
A level or equivalent	0.90 (0.74 – 1.10)	0.30
College or University	0.93 (0.78 – 1.11)	0.45
Chronic disease (n)	1.16 (1.08 – 1.23)	<0.001
Depressive symptoms	1.20 (1.02 – 1.42)	0.032
Cognition	0.76 (0.69 – 0.84)	<0.001
Mobility impairments	1.04 (1.10 – 1.07)	0.003
Social isolation	1.04 (0.92 – 1.18)	0.53
Smoking	1.64 (1.37 - 1.96)	<0.001
Physical activity	0.81 (0.77 – 0.86)	<0.001
Alcohol consumption	1.00 (0.99 – 1.01)	0.59
Fruit and vegetables	0.98 (0.95 – 1.01)	0.12

© 2020 Steptoe A et al. *JAMA Network Open.*
